# The impact of bone marrow sparing on organs at risk dose for cervical cancer: a Pareto front analysis

**DOI:** 10.3389/fonc.2023.1138433

**Published:** 2023-06-28

**Authors:** Sander Kuipers, Jérémy Godart, Anouk Corbeau, Abdul Wahab Sharfo, Sebastiaan Breedveld, Jan Willem Mens, Stephanie de Boer, Remi Nout, Mischa Hoogeman

**Affiliations:** ^1^ Department of Radiotherapy, Erasmus MC Cancer Institute, University Medical Center Rotterdam, Rotterdam, Netherlands; ^2^ Department of Medical Physics and Informatics, HollandPTC, Delft, Netherlands; ^3^ Department of Radiation Oncology, Leiden University Medical Center, Leiden, Netherlands

**Keywords:** VMAT, locally advanced cervical cancer, Pareto front analysis, OAR sparing, bone marrow sparing

## Abstract

**Background and purpose:**

To quantify the increase in bladder and rectum dose of a bone marrow sparing (BMS) VMAT strategy for primary treatment of locally advanced cervical cancer (LACC).

**Materials and methods:**

Twenty patients with stage IB-IVA cervical cancer were selected for this study. The whole Pelvic Bones (PB) was taken as substitute for bone marrow. For every patient, Pareto-optimal plans were generated to explore the trade-off between rectum, bladder, and PB mean dose. The PB mean dose was decreased in steps of 1 Gy. For each step, the increase in rectum and bladder mean dose was quantified. The increase in mean dose of other OAR compared to no BMS was constrained to 1 Gy.

**Results:**

In total, 931 plans of 19 evaluable patients were analyzed. The average [range] mean dose of PB without BMS was 22.8 [20.7-26.2] Gy. When maximum BMS was applied, the average reduction in mean PB dose was 5.4 [3.0-6.8] Gy resulting in an average mean PB dose of 17.5 [15.8-19.8] Gy. For <1 Gy increase in both the bladder and the rectum mean dose, the PB mean dose could be decreased by >2 Gy, >3 Gy, >4 Gy, and >5 Gy for 19/19, 13/19, 5/19, and 1/19 patients, respectively.

**Conclusion:**

Based on the comprehensive three-dimensional Pareto front analysis, we conclude that 2-5 Gy BMS can be implemented without a clinically relevant increase in mean dose to other OAR. If BMS is too dominant, it results in a large increase in mean dose to other OAR. Therefore, we recommend implementing moderate BMS for the treatment of LACC patients with VMAT.

## Introduction

The standard treatment for locally advanced cervical cancer is a combination of external beam radiotherapy (RT) with concurrent cisplatin-based chemotherapy and image-guided brachytherapy (1). This treatment provides high local and pelvic tumor control and cancer-specific survival (2, 3). However, this combination of treatment modalities is associated with a substantial risk of developing hematologic toxicities (HT) grade 2 or higher, e.g. leukopenia and neutropenia (4). In a recent study by Huang et al., the incidence of HT2+ was 69.5% with the standard treatment of locally advanced cervical cancer (LACC) (5) and in the INTERTECC-2 study by Mell et al., the incidence of HT3+ was 31.4% (6). HT can necessitate blood transfusions and lead to an increased risk of infection, missed chemotherapy cycles, or an extension of the treatment time (7, 8). Furthermore, radiation-induced lymphopenia may be associated with lower overall survival (9).

The high incidence of HT is associated with the radiation dose to the pelvic bones and lower spine, in which a large amount of hematopoietically active bone marrow cells are located (10). The irradiation of circulating blood cells in that region might also cause HT (11). Several studies investigated predictors of HT during treatment for LACC, as analyzed in recent reviews (4, 12). Both low and high doses to the pelvic bones were shown to be important in the risk of developing HT (5, 13–15). The evidence from earlier research also suggests that the incidence of HT can be reduced if bone marrow sparing (BMS) is introduced during treatment plan optimization (5, 6, 12, 16). However, the risk of implementing BMS is that it leads to an increase of the dose to other areas of the pelvic region, possibly in organs at risk (OAR) such as the bladder, rectum, and small bowel. A higher dose to these OAR could increase the risk of gastrointestinal and genitourinary toxicities (GI/GU), which are two of the most frequently reported morbidities related to this treatment (2), and could have a clear impact on quality of life (17).

Several treatment planning studies have assessed the impact of BMS (12). A significant increase in GI and GU toxicity grade 2+ as a result of implementing BMS has not been observed (5). However, most studies considered IMRT and did not use the EMBRACE II planning constraints, resulting in a large variation in applied constraints and, consequently, in results (18). Furthermore, all earlier studies have evaluated only a fixed degree of BMS, instead of the full large range of BMS. Therefore, the maximum degree of BMS that can be achieved without clinically significant dose increases for other OARs using VMAT with EMBRACE II planning constraints has not been established.

The aim of this study was to systematically evaluate the trade-off between BMS and dose increases for other OAR for VMAT using the EMBRACE II protocol. To this end, we used automated treatment planning to create three-dimensional Pareto fronts for the mean dose of the bladder, rectum, and pelvic bones. Based on these fronts, we determined the maximum degree of BMS possible without clinically significant dose increases for the other OARs.

## Methods

### Patient data

LACC patients treated at Erasmus MC between December 2019 and January 2021 according to the EMBRACE II protocol were selected for this study (18). All patients underwent an empty and a full-bladder planning CT scan with 2.5 mm slice thickness in a supine position prior to treatment. The minimal field of view of the scans was from 5 cm inferior of the ischial tuberosities to the L1 vertebra. All patients received drinking instructions prior to the full-bladder planning CT (19).

### Normal tissue delineation

All OAR were delineated on the full-bladder planning CT following the EMBRACE II protocol (18). The delineated OAR included the bladder, rectum, sigmoid, bowel bag, femoral heads, and spinal cord. Depending on the level of the target volume, the kidneys, liver, and duodenum were also delineated. The rectum was outlined from 2 cm of the anal canal inferiorly to the recto-sigmoid junction superiorly. The bowel bag (outer extension) was delineated superiorly 2 cm above the planning target volume (PTV). In addition, for this study, the outer contour of the pelvic bones (PB) was taken as a substitute for the bone marrow. PB defined as the outer contour was shown to be most predictive of HT (4). The pelvic bones were delineated from the inferior level of the ischial tuberosities to 2 cm superior to the PTV (20). This includes the femoral heads. Superiorly, the border of the delineation is usually at the L4 for medium-risk patients and at the L1 for high-risk patients. The delineation of the pelvic bones are done by SK (20). All other contours were delineated by radiation oncologists during the regular clinical workflow at the Erasmus MC. All contours were checked afterwards (SK, RN) and, when necessary, adapted to ensure consistency.

### Target delineation

The low-risk Clinical Target Volume (CTV-LR) included the uterus, cervix, gross tumor, parametria, and proximal vagina with 2 cm margin from the GTV. For patients with a uterus movement of >2 cm between the full and empty-bladder CT scans, a plan-of-the-day strategy was applied with a full and an empty-bladder plan (21). In this study, we only considered the full-bladder plan for movers, for which the internal target volume (ITV) was constructed by encompassing the CTV-LR of the half-full bladder to full bladder (22). This CTV-LR for the half-full bladder was created by interpolation between the CTV-LR on the full-bladder CT scan and the CTV-LR on the empty-bladder CT scan. The ITV of non-movers, i.e., a uterus movement of <2 cm, encompassed the CTV-LR position from empty to full bladder and was uniformly expanded with a 0.5 cm margin.

The risk-adapted Clinical Target Volume (CTV-E) consisted of the relevant lymph nodes regions as described in the EMBRACE II protocol (18). The Planning Target Volume (PTV) was constructed by combining the CTV-E and ITV and uniformly expanding this volume by 0.5 cm. The nodal planning target volumes (PTV-N) were constructed following the EMBRACE II protocol (18).

### Pareto-optimal planning

For treatment planning, Erasmus-iCycle was used, our in-house developed algorithm for fully automated multi-criteria treatment plan generation (23). The plans that are generated using this treatment planning system are Pareto optimal (23). This indicates that an objective for such a plan cannot be improved without deteriorating another objective. Examples of such objectives are sufficient target coverage or a low OAR dose. The prescribed dose was 42.75 Gy to 95% of the PTV volume in 25 fractions. The PTV-N received with a simultaneous integrated boost a prescribed dose of 55.0 or 57.5 Gy, depending on the dose that the PTV-N receives during brachytherapy. The plans were created by following the dose constraints of the EMBRACE II protocol (18).

For each patient, a three-dimensional (3D) Pareto front between the rectum, bladder, and PB mean dose was built. A Pareto front is a set of Pareto-optimal plans. In this study, the 3D Pareto front of a patient consisted of four to eight two-dimensional (2D) Pareto fronts showing a trade-off between the bladder and rectum dose. The PB mean dose is constant for each 2D Pareto front. In the representation, these 2D fronts can be thought of as isolines for the PB mean dose. The number of 2D fronts depends on the maximally reachable BMS. Each 2D front consisted of seven Pareto-optimal plans. To build the front for a patient, a reference plan was created without BMS by not using a constraint or objective for the PB in the optimization of the treatment plan. The mean dose of the sigmoid, bowel, kidneys, and duodenum in the following Pareto-optimal plans was constrained to a maximum increase of 1 Gy compared to the reference plan. The first 2D front of a patient had the same mean PB dose as the reference plan, corresponding to 0 Gy BMS, and for each consecutive seven plans for that patient, we decreased the mean PB dose in steps of 1 Gy until the maximum BMS was reached. This is done by making the PB the second most important objective in the wish-list, after the target objective, and decreasing the goal of the objective in steps of 1 Gy. The wish-list used in Erasmus-iCycle is shown and more extensively discussed in **Supplementary Materials**.

For each Pareto front, the mean dose of the PB, bladder, and rectum was compared. These bladder and rectum mean doses were chosen as endpoints because of their anatomical location and relevance regarding treatment-related morbidity (3). For the whole bone, the V_10Gy_, V_20Gy_, V_40Gy_, and the mean dose were chosen as predictors for hematologic toxicity (4).

Our in-house treatment planning system Erasmus-iCycle uses a fluence-based optimization. Furthermore, 20-beams intensity-modulated radiotherapy (IMRT) planning was used to simulate a VMAT planning (24, 25). The beam arrangement consisted of 20 equiangular beams spanning from 0° to 342°. The plans were made for a 6MV flattening beam. To validate the effect of segmentation and the possibility to simulate VMAT with 20-beams IMRT, 5 treatment plans were replanned for 3 patients (15 in total) with a clinical version of the treatment planning system Eclipse (v. 17.0.0). These three patients were randomly chosen from the patients without simultaneous integrated boosts (SIB), as simulating the VMAT plan in Eclipse was only validated in our clinic for patients with one prescribed dose level. Double-arc plans were created with two 358° coplanar arcs. The dose calculation was performed with the Acuros 17.01 algorithm and the optimization with the PO 17.0.1 algorithm. The method of this validation is further discussed in the **Supplementary Materials**.

### Statistical analysis

**Figure 1:**
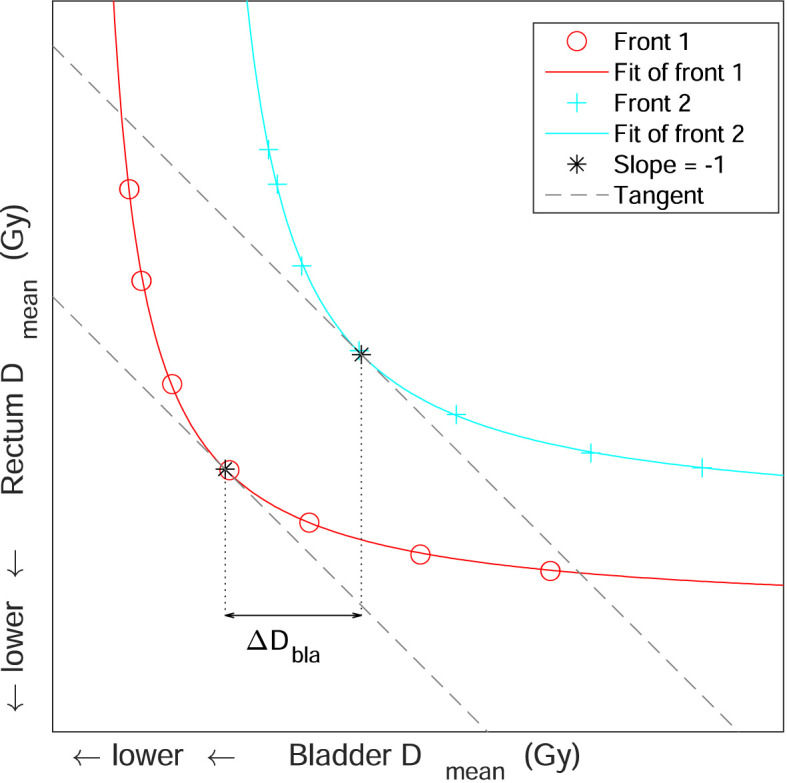
An example of a three-dimensional Pareto front, consisting of two two-dimensional Pareto fronts. The two-dimensional Pareto fronts both consist of seven Pareto-optimal plans and show a trade-off between the bladder and rectum mean dose. The two fronts are fitted and the coordinates where the slope of the fit is equal to -1 is indicated with an asterisk. The tangents of these coordinates, which have a slope of -1, are also shown. The horizontal double arrow shows the difference in bladder mean dose $\text{Δ}D_{Bla}$ between these two coordinates.

The 3D Pareto front of each patient consisted of a set of 2D fronts. Each 2D front showed the trade-off between the bladder and rectum mean dose for a constant PB mean dose. In **Figure 1**, an example of a 3D Pareto front with two 2D fronts, both consisting of seven plans, is shown. To quantify the increase in bladder and rectum dose when decreasing the PB mean dose, we selected one point on each 2D fronts. This point is representative for an equal weight between the bladder and rectum. Next, the difference in dose between these points was calculated for evaluation. For defining the points on the 2D fronts, we fitted each front with the function $D_{Bla}\left(D_{Rec}\right)=\frac{\alpha }{D_{Rec}}+\beta$ , where $D_{Bla}$ and $D_{Rec}$ are the bladder and rectum mean dose and α and β are the fit parameters for the front. Next, the point is defined as the coordinate where the slope is equal to -1, such that the bladder and rectum are equally weighted. The difference in bladder mean dose $\text{Δ}D_{Bla}$ and rectum mean dose $\text{Δ}D_{Rec}$ was then quantified by the difference between these points. In **Figure 1**, the 2D fronts are fitted and the coordinate on both fronts with a slope of -1 is shown.

The Pareto front for each patient consisted of four to eight 2D fronts. The increase for all patients of the bladder and rectum mean dose as a function of the BMS was fitted for multilevel analysis with an exponential mixed-effects regression model. The accurateness of this model is described by the mean-squared error and the log-likelihood.

Statistical analysis was conducted to assess the organ-at-risk (OAR) using various parameters. For the body, we analyzed the V_10Gy_. For the bladder, bowel, rectum, and sigmoid, we examined the V_30Gy_ and V_40Gy_. Additionally, the V_15Gy_ was analyzed for the bowel, the D_mean_ for the kidneys, and the maximum dose (D_max_) for the spinal cord. Regarding the target coverage, we performed statistical analysis for PTV D_95%_, D_50%_, and D_0.1%_. Conformality was evaluated for V_36Gy_ and V_43Gy_, with conformality defined as the ratio of V_36Gy_ or V_43Gy_ of the body to the volume of the PTV. All statistical analysis were conducted at 1, 2, and 3 Gy BMS in reference to 0 Gy BMS with the paired t-test at significance levels p ≤. 05 and p ≤. 001. All statistical analyses were performed in Matlab 2012b.

## Results

Twenty patients with FIGO 2018 stage IB-IVA cervical cancer were selected for this study. The mean age was 49 years (range 30 - 77). 15/20 patients received simultaneous integrated boost (SIB) to pelvic lymph nodes and in 2/20 patients were identified as high-risk patients and had the paraaortic region included in the CTV-E. The other patients were all medium-risk patients.

For the twenty patients, we generated in total 987 plans. Of the cohort, 19 of the 20 patients were evaluable for analysis, which corresponds to 931 Pareto-optimal plans. One patient was excluded because of the bladder volume having a large overlap with the PTV (104/118 cc) on the full-bladder CT scan, making it impossible to spare the bladder without deteriorating target coverage. In **Supplementary Figure S3**, the plans with no BMS are shown for all twenty patients and the Pareto front for the excluded patient is shown in cyan. After optimization, all treatment plans satisfied the constraints of the EMBRACE II protocol, however, after calculating the dose grid at CT resolution from the optimization grid, 842/931 (90.4%) satisfied the constraints (18). When the D_max_ and D_min_ were based on D_99.8%_ and D_0.2%_ instead of D_99.9%_ and D_0.1%_, 931/931 (100%) plans satisfy the constraints. The average conformality for the 36 Gy volume, defined as V_36Gy_/Volume of PTV, is 1.50 ± 0.06 and for the 43 Gy volume is 1.07 ± 0.02.

**Figure 2:**
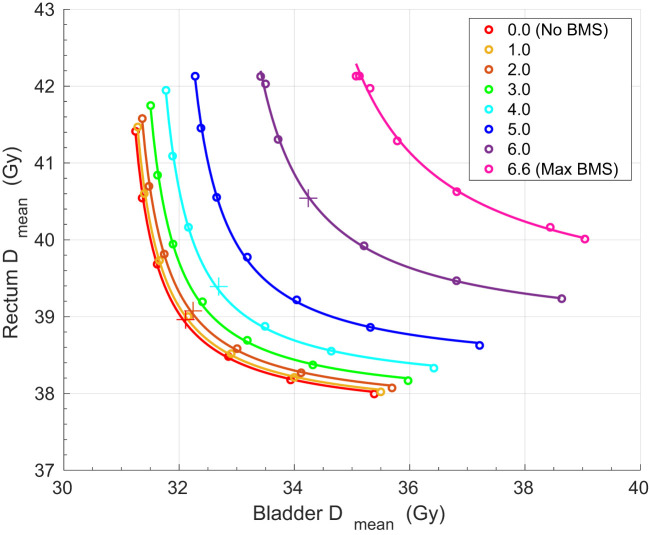
The three-dimensional Pareto front for one patient. The Pareto front consists of 56 Pareto-optimal plans. 0 Gy sparing corresponds to a mean whole bone dose of 24.6 Gy and 6.6 Gy is the maximum BMS. The plans shown in **Figure 3** are indicated with a plus-sign.

**Figure 3:**
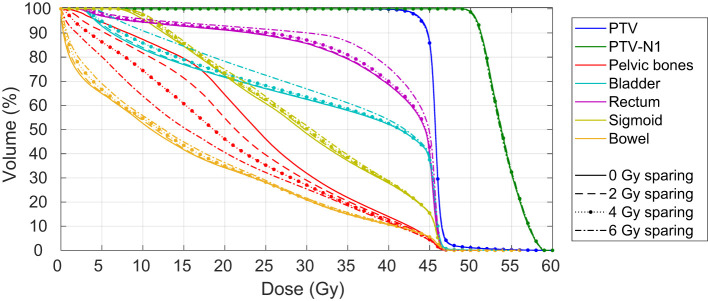
Dose volume histogram of the PTV, PTV-N1, pelvic bones, bladder, rectum, sigmoid and bowel for 0, 2, 4, and 6 Gy bone marrow sparing for one patient.

**Figure 4:**
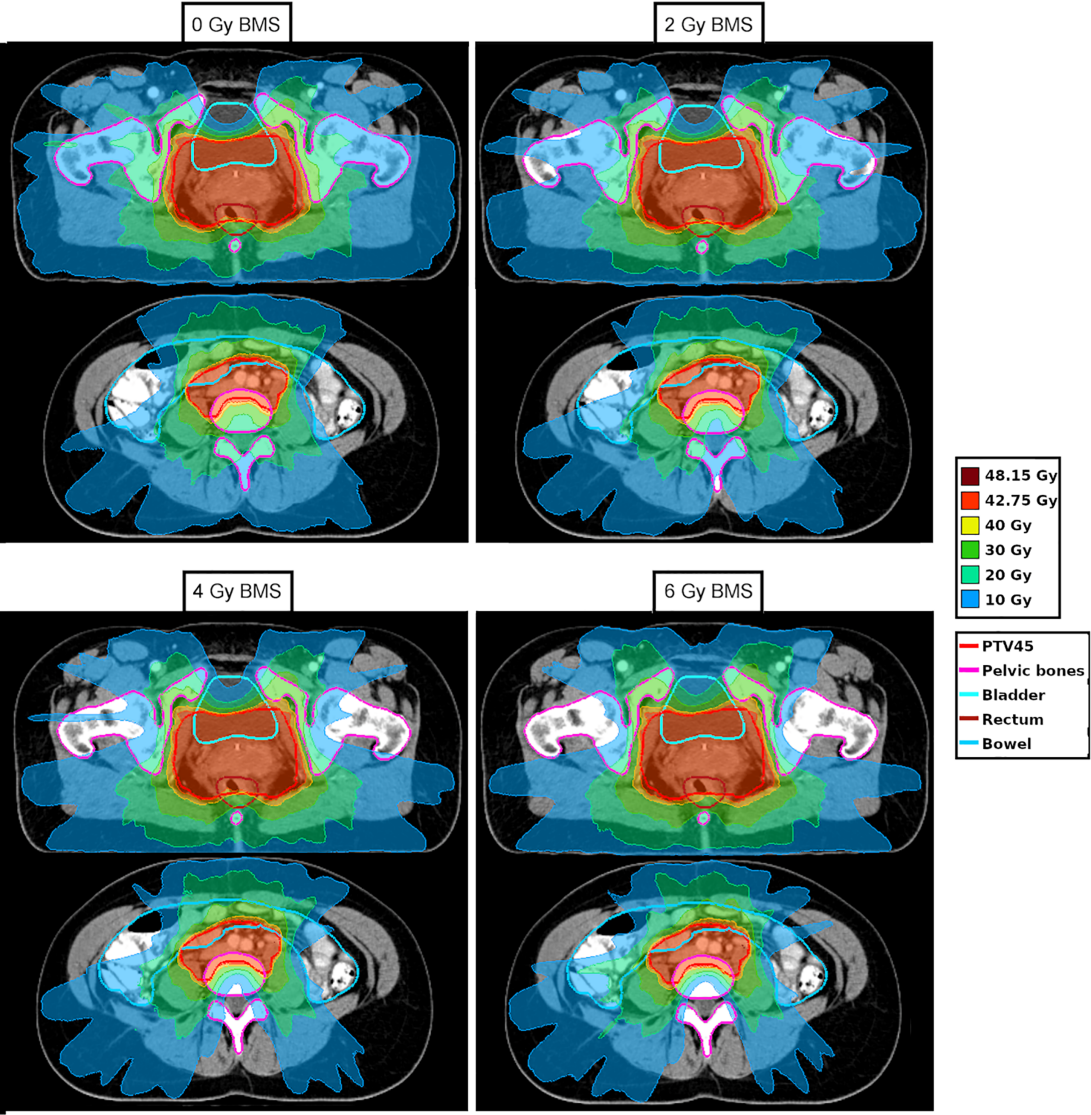
Dose distribution for one patient for 0, 2, 4, and 6 Gy bone marrow sparing (BMS). The contour of the pelvic bones is shown in pink.

**Figure 2** shows the full three-dimensional Pareto front of patient 17. The relatively large reduction in bone marrow dose for this patient helps to visualize the impact of BMS. For this patient, the mean PB dose could be reduced by up to 6.6 Gy, from 24.6 Gy to 18.0 Gy. The reduction of the PB dose and the impact of the dose on the other OAR is visualized in the DVH in **Figure 3** for four different plans. The corresponding dose distributions are shown in **Figure 4**. The dose reduction in the PB is clearly visible while the dose increase in the other OAR only starts to be evident at 6 Gy BMS.

The average [range] mean dose in the PB for all 19 patients with no BMS was 22.8 [20.7 - 26.2] Gy. When maximum BMS was applied, the average reduction in mean PB dose was 5.4 [3.0-6.8] Gy resulting in a mean PB dose of 17.5 [15.8-19.8] Gy. The average PB V_10Gy_, V_20Gy_, and V_40Gy_ are shown in **Supplementary Table S2** for 0, 1, 2, and 3 Gy BMS. The conformality index and different dosimetric parameters for OAR and the target coverage are shown in **Supplementary Table S3** for 0, 1, 2, and 3 Gy BMS.

**Figure 5:**
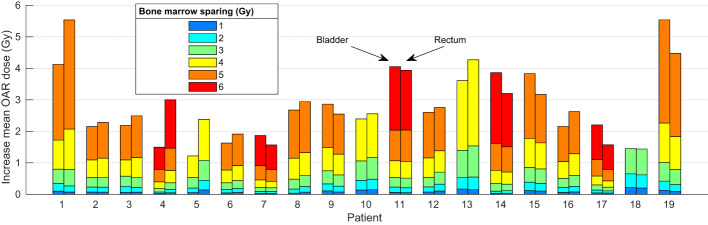
The increase in bladder (left bar) and rectum (right bar) mean dose for implementing 1,2, 3, 4, 5, or 6 Gy BMS for each patient.

**Figure 5** shows the increase in the bladder $\text{Δ}D_{Bla}\left(BMS\right)$ and rectum mean dose $\text{Δ}D_{Rec}\left(BMS\right)$ for each patient as a function of the bone marrow sparing *BMS*. Patients 4 and 7 are the patients with the paraaortic region included. If <1 Gy increase in both the bladder and rectum mean dose is chosen as a clinically acceptable increase, the PB mean dose could be decreased by >1 Gy, >2 Gy, >3 Gy, >4 Gy, and >5 Gy for 19/19, 19/19, 13/19, 5/19, and 1/19 patients, respectively.

**Figure 6:**
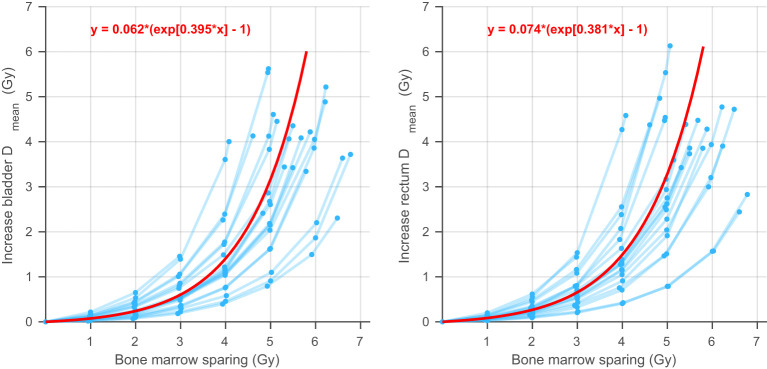
A visualization of the exponential multilevel analysis. Each blue line corresponds to one patient and shows the average increase in bladder (left) or rectum (right) mean dose as a function of the bone marrow sparing.

The increase in bladder and rectum mean dose as a function of BMS is shown in **Figure 6**. Each black line represents a different patient. All patients show a superlinear increase which can be fitted by an exponential function. The fits of an exponential multilevel analysis are indicated with a red line. The fits are given by $Di_{Bla}\left(BMS\right)=0.062*\left(exp\left[0.395*BMS\right]-1\right)$ and $Di_{Rec}\left(BMS\right)=0.074*\left(exp\left[0.381*BMS\right]-1\right)$ with a mean-squared error of 0.0030 and 0.0011 and a log-likelihood of 100.4 and 144.5, respectively.

In **Supplementary Figure S1**, the dose volume histogram for one patient is shown for 0 and 3 Gy BMS for the Erasmus-iCycle and the segmented VMAT plans. In **Supplementary Figure S2**, the increase in bladder and rectum dose as a function of BMS of the segmented VMAT plans is shown and compared to the corresponding Erasmus-iCycle plans. All segmented plans that were constructed using the clinical treatment planning system Eclipse, fulfil the EMBRACE II constraints and have an adequate target coverage. The plans show a superlinear increase similar to the Erasmus-iCycle plans without impacting the target coverage and the clinical constraints.

## Discussion

The purpose of this study was to analyze the relation between pelvic bones, bladder, and rectum dose for constant target coverage. This study indicates that the dose to the bladder and rectum increases superlinearly when the pelvic bones dose decreases. Moderate BMS results in a small increase in bladder and rectum dose. It also showed that the PB dose could be reduced further for some patients, but at some point, BMS leads to a substantial increase in dose to the bladder and rectum. It depends on the patient anatomy what a reasonable level of BMS is. These results were obtained after a systematic analysis using automatic planning (Erasmus-iCycle) and were validated in a clinical planning system (Eclipse).

Reducing the PB dose is important as it has been reported that hematologic toxicity is associated with the dose to the PB (9–11). Besides HT, reducing the dose to PB may decrease the risk of (insufficiency) fractures, which is another frequently reported toxicity after pelvic radiotherapy (26). The dosimetric cut-off values for PB to reduce HT imposed by earlier literature are V_10Gy_ < 75-95% (13, 20, 27), V_20Gy_ < 65-80% (13, 28–30), and V_40Gy_ < 28-37% (5, 15). The V_10Gy_ greatly benefits from BMS as the constraint V_10Gy _< 75% was only accomplished in 2/133 (1.5%) patients for the no-BMS plans and 84/133 (63.1%) for the 3 Gy BMS plans. The constraint V_20Gy _< 65% is reached in 126/133 (94.7%) plans with no BMS and in all plans for ≥1 Gy BMS. The high dose constraint V_40Gy_ < 28% is already achieved by all no-BMS plans. However, it is unclear if these proposed constraints could be further improved, thus benefits in further reducing the dose can be expected.

The results of this study indicate moreover that BMS planning techniques should be implemented with care. The sparing of the bone marrow is shown to result in a large increase of the bladder and/or rectum dose in case the BMS is too dominant in the chosen planning strategy. For some patients, 5 Gy BMS increases both the bladder and rectum mean dose with more than 4 Gy. The clinical impact of this change in bladder, rectum, and whole bone dose on GI, GU, and hematologic toxicity is however not well known. Further research must be done on identifying the toxicity effect of dosimetric changes in the OAR, to find the optimal balance between OAR sparing during the treatment planning process.

Another interesting result of this study is that the volume of the body exposed to 10 Gy (body V_10Gy_) decreases significantly when BMS is implemented. For 3 Gy BMS the body V_10Gy_ decreases by 537 cc in comparison to no BMS. The volume of the body exposed to radiation was also shown to be significantly correlated with HT (31). Furthermore, our findings indicate that the high-risk patients show a larger potential for benefiting in BMS. **Figure 5** illustrates that patient 4 and 7, classified as high-risk, demonstrate relatively large maximum BMS for a small increase in OAR dose. However, due to the limited number of patients included in this study, an analysis on the Pareto fronts for different classifications could not be conducted. Future studies with larger patient cohorts may provide further insights in this regard.

Based on the results of this study, we advocate the inclusion of PB as an OAR in clinical routine for (VMAT) treatment planning of cervical cancer, but the weight of the PB planning objective should be kept relatively low in comparison to the objectives of other OAR. The precise method to do this differs per treatment planning system. With the use of a low weight for PB sparing, the initial no-BMS plan does not have to be generated to create a baseline in PB mean dose, as was done for this study, and the mean dose of the rectum and bladder does not increase substantially. The outcomes of the validation of the Pareto fronts, discussed in the **Supplementary Materials**, show that these findings can be reproduced with a clinical treatment planning system and are feasible in the clinic. We foresee no reasons why the results for other tumor sites in the pelvic region would be substantially different. This is therefore a broad recommendation applicable to VMAT treatments of pelvic tumor sites with a high incidence of HT.

Several treatment planning studies have compared no-BMS with BMS plans, however, most studies considered IMRT and there is a large variation in the planning dose constraints and the results. Furthermore, all earlier studies have taken only a fixed degree of BMS into account, whereas, with the Pareto front analysis, we were able to show the dosimetric impact on the rectum and bladder for multiple degrees of BMS with a very small to no change in the target coverage and still fulfilling the planning constraints of the EMBRACE II protocol.

This study demonstrates the advantage of using a systematic approach to introduce a new OAR in a clinical treatment planning strategy. With this meticulous methodology, the effect of different degrees of sparing a new OAR can be quantified. To the best of our knowledge, the present study is the first to investigate the introduction of a new OAR with this methodology. This study shows that Pareto front analysis is an interesting method to determine which objectives should be used in the clinic. While we investigated Pareto fronts for the sparing of the PB for the treatment of cervical cancer with VMAT, the technique is also applicable for other tumor sites, OAR, and treatment modalities. With the same methodology of BMS, future studies can investigate the possibilities of BMS for proton therapy (32).

## Conclusion

Based on a comprehensive Pareto front analysis, we conclude that it is possible to decrease the pelvic bones mean dose by 2-5 Gy without increasing the dose to other OAR with a clinically relevant amount (>1 Gy) and that the dose to the bladder and rectum increases superlinearly when decreasing the bone marrow dose. Excessive BMS could however result in large increases in OAR dose. Therefore, we recommend implementing moderate bone marrow sparing for VMAT treatment planning of patients with LACC.

## Data availability statement

The datasets presented in this article are not readily available because ethical and privacy restrictions. Requests to access the datasets should be directed to Sander Kuipers, s.c.kuipers@erasmusmc.nl.

## Ethics statement

The studies involving human participants were reviewed and approved by Institutional Review Board of the Erasmus University Medical Center Rotterdam. The patients/participants provided their written informed consent to participate in this study.

## Author contributions

JG, MH, SdB and RN contributed to the original design of the study. JM and JG supervised the data acquisition. SK, AS and SB built the model. SK did the statistical analysis. JG, SK, AC, SdB, RN and MH contributed to the final design of the study. SK wrote the first draft of the article. All authors contributed to manuscript revision. All authors contributed to the article and approved the submitted version.
