# Supplementary material for: The impact of bone marrow sparing on organs at risk dose for cervical cancer: a Pareto front analysis
Source: Front Oncol. 2023 Jun 28;13:1138433. doi: 10.3389/fonc.2023.1138433 (PMC10338058; doi:10.3389/fonc.2023.1138433)
Supplement: Supplementary file 1 [file DataSheet_1.docx]

## Supplementary Material

The wish-list for creating the Pareto fronts

The optimization in Erasmus-iCycle is based on a wish-list. This wish-list consists of constraints, which will always be met, and treatment objectives with assigned priorities. The objectives are planning aims which need to be met as closely as possible (or supersede, if possible). The wish-list used for creating the Pareto front is shown in Table S1. The constraints *C_1_* to *C_10_* in the wish-list followed from the EMBRACE II protocol [15]. Furthermore, the constraints *C_11_* to *C_15_* and the objective *O_6_* are to keep the dose conformal with ring and shell structures around the target. The mean dose of the bowel, sigmoid, kidneys and duodenum is constrained to a maximum of 1 Gy increase compared to the base plan with the variables *A_1_* to *A_5_* in the constraints *C_16_* to *C_20_*. The variable *G* in the objective *O_2_* defines the degree of BMS in the optimization. For the base plan, this objective is inactive. To create a Pareto front showing trade-off between the bladder and rectum mean dose, seven plans are created with varying weights for the objective *O_3_* and *O_4_*. The relative weight for the seven plans in a Pareto front is given by $\frac{W_{Bla}}{W_{Rec}}=\{{2.3}^{-3},{2.3}^{-2},{2.3}^{-1},{2.3}^{0},{2.3}^{1},{2.3}^{2},{2.3}^{3}\}$.

Supplementary Table S1 The Erasmus-iCycle wish-list used for automated IMRT/VMAT plan generation for cervical cancer patients. *PTV = planning target volume; ITV = internal target volume; CTV = clinical target volume; CTV-HR = high risk CTV; LTCP = logarithmic tumor control probability.*

| *Constraints* | | **Structure** | **Type** | **Limit** |  |
| --- | --- | --- | --- | --- | --- |
|  | |  |  |  |  |
| *C_1_*  *C_2_*  *C_3_*  *C_4_*  *C_5_*  *C_6_*  *C_7_*  *C_8_*  *C_9_*  *C_10_*  *C_11_*  *C_12_*  *C_13_*  *C_14_*  *C_15_*  *C_16_*  *C_17_*  *C_18_*  *C_19_*  *C_20_* |  | CTV-N  PTV-N  PTV-N  ITV45  Body  Bowel  Sigmoid  Spinal Cord  Femoral Heads  CTV-HR + 10 mm  PTV45 ring 20-30 mm  PTV45 ring 30-40 mm  PTV45 ring >40 mm  PTV45 shell 3 mm  PTV-N shell 6 mm  Bowel  Sigmoid  Kidney Left  Kidney Right  Duodenum | Minimum  Minimum  Maximum  Minimum  Maximum  Maximum  Maximum  Maximum  Maximum  Maximum  Maximum  Maximum  Maximum  Maximum  Maximum  Mean  Mean  Mean  Mean  Mean | 0.95 x 55/57.5 Gy  0.90 x 55/57.5 Gy  1.07 x 55/57.5 Gy  0.95 x 45 Gy  1.07 x 45 Gy  1.05 x 45 Gy  1.05 x 45 Gy  1.05 x 45 Gy  1.00 x 45 Gy  1.03 x 45 Gy  0.63 x 45 Gy  0.53 x 45 Gy  0.43 x 45 Gy  0.96 x 45 Gy  1.10 x 45 Gy  *A_1_*  *A_2_*  *A_3_*  *A_4_*  *A_5_* |  |
| *Objectives* | |  |  |  |  |
|  | **Priority** | **Structure** | **Type** | **Limit** | **Weights** |
| *O_1_* | 1 | PTV45 | LTCP |  | 1 |
| *O_2_* | 2 | Pelvic bones | Mean | *G* | 1 |
| *O_3_* | 3 | Bladder | Mean | 0.0 Gy | *W_Bla_* |
| *O_4_* | 3 | Rectum | Mean | 0.0 Gy | *W_Rec_* |
| *O_5_* | 4 | Skin | Maximum | 4.5 Gy | 1 |
| *O_6_* | 4 | PTV45 ring 0-10 mm | Maximum | 22.5 Gy | 1 |
| *O_7_* | 5 | Bowel | Mean | 10.0 Gy | 1 |
| *O_8_* | 6 | Kidney Left | Mean | 5.0 Gy | 1 |
| *O_9_* | 6 | Kidney Right | Mean | 5.0 Gy | 1 |
| *O_10_* | 7 | Sigmoid | Mean | 25.0 Gy | 1 |
| *O_11_* | 8 | Spinal Cord | Maximum | 22.5 Gy | 1 |
| *O_12_* | 9 | Femoral Heads | Maximum | 22.5 Gy | 1 |
|  | | | | |  |

**Validation with a clinical treatment planning system**

All 987 VMAT plans for the 3D Pareto fronts were planned with the in-house treatment planning system (TPS) Erasmus-iCycle. This TPS uses only fluence-based optimization. Furthermore, 20-beams IMRT was used as a substitute for VMAT. To validate if our findings were reproducible with VMAT after segmentation, we replanned 15 plans in the clinical TPS Eclipse (v. 17.0.0). For three patients, the Erasmus-iCycle plans with a bone marrow sparing of 0, 1, 2, 3, and 4 Gy and an equal weighting on the bladder and rectum objective were reproduced with Eclipse by an experienced planner (AS). For the reproduction, DVH parameters of the Erasmus-iCycle plans and the EMBRACE II constraints are imported into Eclipse. The Halcyon^TM^ multileaf collimator system was used with a maximal size of 28x28 cm^2^ [30]. The treatment plans were planned for 6 MV with the FFF beam. The focus was on reproducing the mean dose values of the OAR, on achieving an adequate target coverage, and on fulfilling the EMBRACE II constraints. Figure S1 presents a comparison of the DVH between Eclipse and iCycle plans. Small deviations are observed in the DVH parameters between the Erasmus-iCycle and Eclipse plans. The segmented VMAT plans from the Eclipse TPS exhibited slightly worse target coverage. However, this does not impact the comparison, as the target coverage remained consistent for multiple degrees of BMS, and all clinical constraints were met.

**Supplementary Figure S1:** Dose volume histogram of the PTV, pelvic bones, bladder, rectum, sigmoid, bowel, and the patient for Erasmus-iCycle and Eclipse plans. The dose volume histogram is shown for 0 and 3 Gy BMS.

**Supplementary Figure S2:** The increase in bladder (left) and rectum (right) mean dose as a result of the bone marrow sparing. A comparison is made between five Erasmus-iCycle plans (solid) and five segmented VMAT Eclipse plan (dashed) for three patients.

**Supplementary Figure S3:** The Pareto fronts with no BMS for all twenty patients. Each Pareto front consists of seven Pareto-optimal plans. The Pareto front indicated in cyan is excluded from the analysis.

**Supplementary Table S2:** The average dosimetric parameters [range] for the PB for different magnitudes of BMS. *PB = Pelvic bones; BMS = bone marrow sparing*

| **Dosimetric parameters** | **0 Gy BMS** | **1 Gy BMS** | **2 Gy BMS** | **3 Gy BMS** |
| --- | --- | --- | --- | --- |
| PB D_mean_ (Gy) | 22.81 [20.70 – 26.26] | 21.82 [19.71 – 25.26] | 20.82 [18.71 – 24.26] | 19.83 [17.71 – 23.28] |
| PB V_10Gy_ (%) | 83.0 [74.6 – 93.3] | 80.0 [71.8 – 91.6] | 76.7 [68.5 – 87.9] | 73.0 [64.6 – 83.8] |
| PB V_20Gy_ (%) | 57.3 [49.0 – 66.9] | 53.0 [44.7 – 62.2] | 48.5 [40.7 – 58.2] | 44.7 [37.0 – 54.5] |
| PB V_40Gy_ (%) | 12.0 [9.4 – 16.3] | 11.6 [9.0 – 15.8] | 11.1 [8.6 – 15.3] | 10.6 [8.1 – 14.7] |

**Supplementary Table S3:** The mean dosimetric parameters [range] for the OAR and the target volumes and the mean conformality index [range] for different magnitudes of BMS. The significance is calculated for 1, 2, and 3 Gy BMS with 0 Gy BMS as the reference. *BMS = bone marrow sparing; CI = conformality index.*

| **Dosimetric parameters** |  | **0 Gy BMS** | **1 Gy BMS** | **2 Gy BMS** | **3 Gy BMS** |
| --- | --- | --- | --- | --- | --- |
| Bladder | D_mean_ (Gy) | 33.0 [23.9 – 43.0] | 33.1 [24.0 – 43.1]** | 33.3 [24.3 – 43.2]** | 33.7 [24.9 – 43.4]** |
|  | V_30Gy_ (%) | 64.9 [42.2 – 98.3] | 65.1 [42.3 – 98.3]* | 65.6 [42.9 – 97.8]** | 66.6 [43.8 – 97.5]** |
|  | V_40Gy_ (%) | 49.8 [29.9 – 82.9] | 49.8 [30.0 – 83.0]** | 50.0 [30.2 – 83.1]** | 50.3 [30.7 – 83.8]** |
| Rectum | D_mean_ (Gy) | 30.6 [16.6 – 41.2] | 30.7 [16.7 – 41.3]** | 30.9 [16.8 – 41.4]** | 31.3 [17.3 – 41.6]** |
|  | V_30Gy_ (%) | 58.4 [21.9 – 91.7] | 58.6 [22.0 – 91.9]** | 59.2 [22.3 – 92.1]** | 60.5 [23.1 – 92.5]** |
|  | V_40Gy_ (%) | 38.8 [11.4 – 79.0] | 39.0 [11.4 – 78.9]** | 39.4 [11.6 – 79.4]** | 40.4 [12.1 – 80.2]** |
| Bowel | D_mean_ (Gy) | 17.0 [13.8 – 23.2] | 17.1 [14.0 – 23.3]** | 17.3 [14.2 – 23.5]** | 17.5 [14.4 – 23.7]** |
|  | V_30Gy_ (cc) | 264 [84 – 619] | 264 [83 – 620]* | 266 [83 – 623]* | 267 [83 – 626]** |
|  | V_40Gy_ (cc) | 137 [32 – 371] | 137 [32 – 372] | 137 [32 – 373]* | 138 [32 – 373]* |
| Sigmoid | D_mean_ (Gy) | 36.6 [15.6 – 45.3] | 36.7 [14.5 – 45.3] | 36.7 [13.9 – 45.3] | 36.7 [14.0 – 45.3] |
|  | V_30Gy_ (%) | 76.1 [20.5 – 100.0] | 76.2 [20.5 – 100.0]* | 76.3 [20.3 – 100.0]* | 76.4 [20.0 – 100.0] |
|  | V_40Gy_ (%) | 59.1 [10.3 – 99.7] | 59.2 [10.3 – 99.7]* | 59.2 [10.1 – 99.7]* | 59.3 [9.9 – 99.7] |
| Kidney L | D_mean_ (Gy) | 1.19 [0.00 – 5.75] | 1.23 [0.00 – 5.66] | 1.26 [0.00 - 5.64] | 1.28 [0.00 – 5.70] |
| Kidney R | D_mean_ (Gy) | 0.85 [0.00 – 5.30] | 0.87 [0.00 – 5.54] | 0.88 [0.00 – 5.68] | 0.89 [0.00 – 5.80] |
| Body | V_10Gy_ (L) | 10.77 [7.30 – 16.62] | 10.62 [7.15 – 16.47]** | 10.45 [7.00 – 16.25]** | 10.23 [6.80 – 15.96] ** |
| Spinal cord | D_max_ (Gy) | 21.6 [11.4 – 30.0] | 18.5 [8.2 – 29.4]** | 14.96 [6.7 – 27.5]** | 12.17 [5.0 – 26.0]** |
| PTV45 | D_95%_ (Gy) | 44.1 [43.8 – 44.3] | 44.1 [43.8 – 44.3]** | 44.1 [43.8 – 44.3]** | 44.1 [43.8 – 44.3]** |
|  | D_50%_ (Gy) | 45.6 [45.4 – 45.7] | 45.6 [45.4 – 45.7] | 45.6 [45.4 – 45.7] | 45.6 [45.4 – 45.7] |
|  | D_0.1%_ (Gy) | 47.7 [47.4 – 48.1] | 47.7 [47.4 – 48.1] | 47.7 [47.4 – 48.1] | 47.7 [47.4 – 48.1] |
| CI | V_36Gy_ | 1.51 [1.42 – 1.67] | 1.50 [1.41 – 1.67]** | 1.50 [1.41 – 1.67]** | 1.50 [1.40 – 1.67]** |
|  | V_43Gy_ | 1.08 [1.04 – 1.12] | 1.07 [1.04 – 1.12]** | 1.07 [1.03 -1.12]** | 1.07 [ 1.02 – 1.13]** |

** = P < 0.05; ** = P < 0.001*
